# Lump on Back: A Rare Case of Parosteal Lipoma of Scapula

**DOI:** 10.1155/2014/169157

**Published:** 2014-09-22

**Authors:** Ankit Balani, Ashwini Sankhe, Tilak Dedhia, Maunil Bhuta, Narayan Lakhotia, Jagir Yeshwante

**Affiliations:** Department of Radiology, Lokmanya Tilak Municipal Medical College and Lokmanya Tilak Municipal General Hospital, Sion, Mumbai 400022, India

## Abstract

Lipomas are benign tumors of mature adipose tissue which can occur in subcutaneous, intramuscular, intermuscular, parosteal, and intraosseous compartments. Parosteal lipoma is a rare type of lipoma, accounting for less than 0.1% of primary bone neoplasms and 0.3% of all lipomas. Parosteal lipomas commonly arise in the femur and extremities. Around 150 cases have been reported in English literature with scapula being a rare site of involvement. They are known to be associated with underlying bony changes like focal cortical hyperostosis, pressure erosion of the underlying bone, and bowing deformity or with underlying osteochondroma. We report a rare case of a parosteal lipoma arising in the scapula with a bony excrescence in a 38-year-old male.

## 1. Case Report

A 38-year-old male presented with complains of a painless swelling gradually increasing in size over the left side of upper back for 3 years. There was no history of any neurological deficit or fever. On clinical examination, an approximately 6 × 5 cm sized mass was palpated adjacent to inferior margin of left scapula. The swelling was soft, nontender, and easily compressible with no evidence of increased local temperature. Distal pulse and neurological examination were normal.

**Figure 1:**
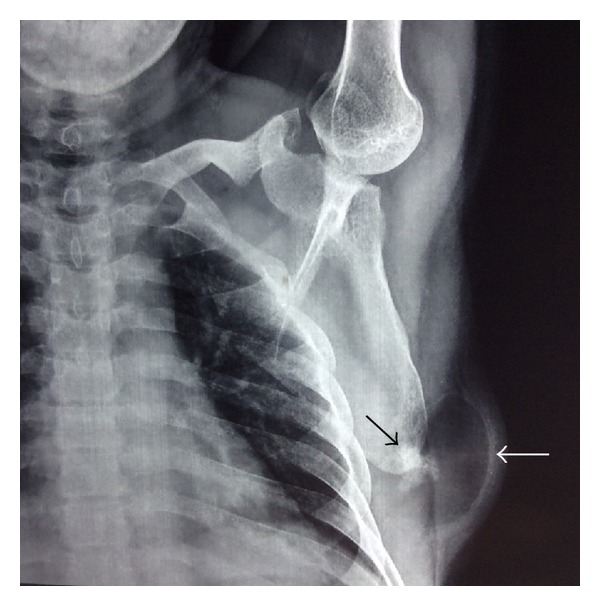
Plain radiograph of left shoulder joint showing a radiolucent lesion (white arrow) with underlying irregular osseous protuberance (black arrow) arising from the inferior tip of scapula.

Plain radiograph of left shoulder joint revealed an irregular osseous protuberance seen contiguous with inferior part of lateral border of left scapula with overlying well-circumscribed radiolucent lesion (Figure 1).

**Figure 2:**
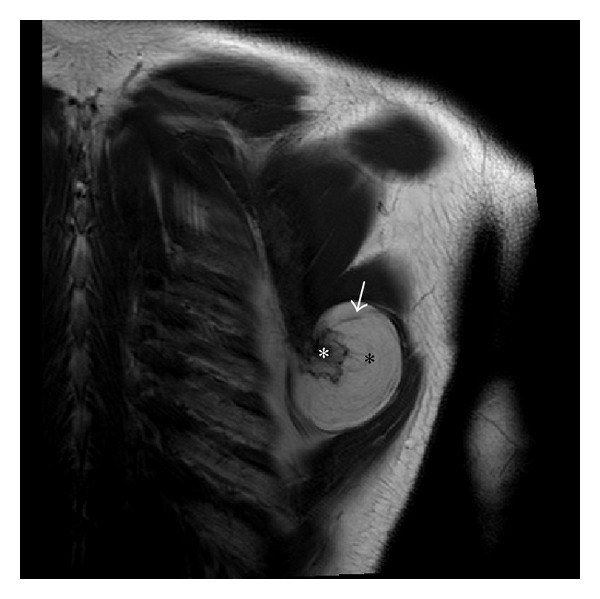
T1 fast spin echo coronal image of left shoulder joint showing well-defined, juxtacortical, and hyperintense lesion (black asterisk) with the multilobulated hypointense bony excrescence (white asterisk) and several thin low signal striations (white arrow) noted arising from inferior border of left scapula.

**Figure 3:**
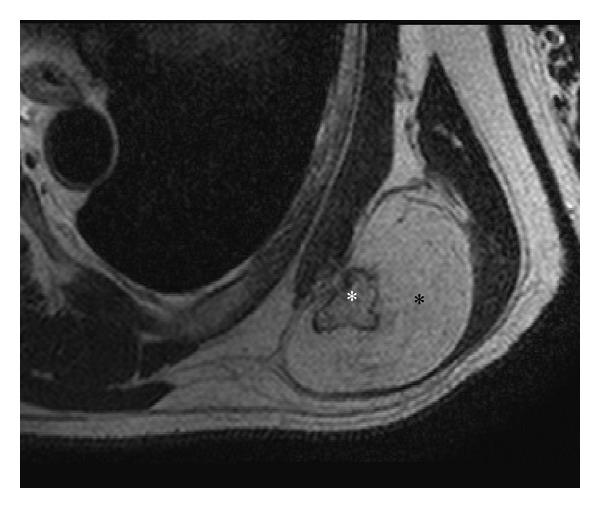
T2 fast spin echo axial image showing well-defined hyperintense lesion (black asterisk) with the multilobulated hypointense bony excrescence (white asterisk) arising from inferior border of left scapula.

**Figure 4:**
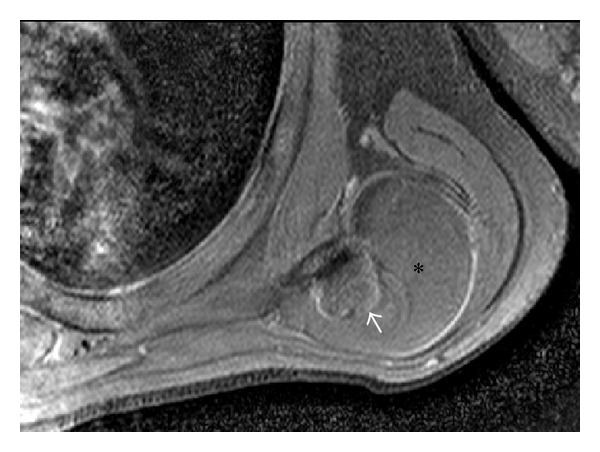
T1 fat saturated precontrast axial image showing suppression of the T1 hyperintensity suggesting fat component (black asterisk) with bony excrescence (white arrow).

**Figure 5:**
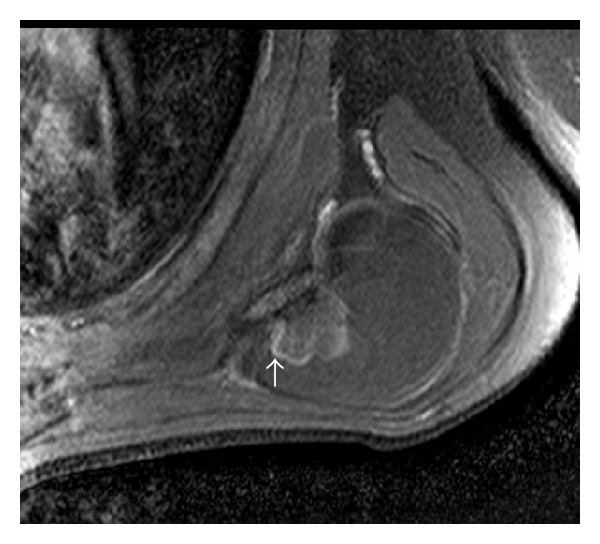
T1 fat saturated postcontrast axial image showing enhancement of interface between the bony protuberance and the lipid component of the lesion suggesting the fibrous tissue rim (white arrow).

To characterize the lesion and define the compression of adjacent neurovascular bundle, plain and contrast enhanced magnetic resonance imaging (MRI) was performed on 3T Achieva Philips MRI scanner which revealed a well-defined, multilobulated, and juxtacortical bony excrescence measuring 2 × 1.7 cm in size adjacent to inferior margin of left scapula showing no contiguity with marrow space of scapula differentiating it from an osteochondroma. There was overlying 4.5 × 5.5 × 6.0 cm (anteroposterior × mediolateral × superinferior) well-defined T1 and T2 hyperintense lesion, which was suppressed on fat saturated imaging (Figures 2, 3, and 4). There was mild enhancement of interface between the bony protuberance and the lipid component of the lesion suggesting the fibrous tissue rim (Figure 5). There were no findings of neurovascular bundle compression. The diagnosis of parosteal lipoma with bony excrescence was made.

The patient refused complete excision of mass which is treatment of choice.

## 2. Discussion

Lipomas are the most common benign mesenchymal tumors that usually arise in soft tissues. Parosteal lipoma is a rare benign tumor of adipose tissue arising from mesenchymal cells of periosteum [1]. Initially called “periosteal lipoma,” the lesion was renamed “parosteal lipoma” to emphasize that periosteum does not contain fat cells [2]. More than 50% of people are about and over the age of 40 years and are aware of the lesion for years [3]. Most common sites are femur followed by proximal radius. Rarely these lesions have been reported arising from scapula, clavicle, ribs, pelvis, metacarpals, metatarsals, mandible, and skull [2]. They show similar features on histopathology as those of the commonly occurring soft tissue lipomas. Even the cytogenetic evidence suggests a common histopathogenesis for both of them.

There are various ways in which a parosteal lipoma occurs depending on degree of chondroid modulation and endochondral ossification (Table 1). The focal hyperostotic change consists of immature periosteal new bone and is the most common finding in the underlying bone as seen in our case.

On radiographs, a parosteal lipoma is a well-defined area of lucency located adjacent to a long bone. Parosteal lipomas may have underlying bony alterations, mostly hyperostotic reactive changes presenting as fine linear densities, calcification, cortical thickening or undulation or frank excrescences of bone, cortical bowing (in patients with growing bones), or smooth cortical erosions, or underlying osteochondroma [3].

On computed tomography, parosteal lipomas have fat density lesion with an osseous lobulated component adherent to the surface of the adjacent bone. Osseous excrescences may be present, but they lack the contiguity of the marrow space with the underlying bone differentiating them from an osteochondroma. Computed tomography characterizes degree of septation and defines relationship of mass with underlying cortex which is important for surgical planning [2].

On MRI, the tumor is identified as a juxtacortical mass with signal intensity identical to that of fat, regardless of pulse sequence with occasional low-signal-intensity strands on T1 in the lesion, corresponding to fibrovascular strands that are commonly found in lipomatous lesions. These strands can be differentiated from those of well-differentiated liposarcoma, as these are thin and lack postcontrast enhancement [4]. T1 and T2 hypointense areas represent osseous components. MRI highlights adjacent muscular atrophy and compression of adjoining neurovascular bundles. Increased striations of fat in the affected muscle are caused by associated nerve entrapment [5]. This finding is best appreciated on T2-weighted images because of the decreased signal intensity of normal muscle relative to fat. Parosteal lipomas are known to cause nerve compression as there have been previous reports of radial, sciatic, ulnar, and median nerve involvement. Interestingly, the anchoring effect of the attachment site at the bone may predispose these parosteal lipomas to mass effect and nerve impingement, as opposed to the pattern of soft tissue lipomas, which expand along the path of least resistance [2]. Postcontrast imaging may reveal patchy nodular enhancement at the interface of the mass and the bony protuberance, raising the possibility of reactive soft tissue change.

In our case, bony protuberance and surrounding juxtacortical benign lipoma were seen in the inferior surface of the left scapula, with underlying cortical hyperostosis, suggestive of the typical findings of parosteal lipoma well appreciated in MR imaging. The exclusion of medullary continuity between bony protuberance and the adjacent bone, which is the differential diagnostic clue from osteochondroma, was also well appreciated in the precontrast T1-weighted images. Additionally, no findings related with nerve compression were detected in MR imaging, providing confidence to the clinical examination results.

Complete excision of the mass is treatment of choice. Prognosis is good with no recurrence postoperatively. Majority of parosteal lipomas have been reported to have no malignant potential and thus can be followed conservatively.

## 3. Conclusion

Parosteal lipoma is a rare benign mesenchymal primary tumor arising from periosteum. Imaging plays an important role in diagnosis, characterization of the lesion with MRI evaluating the effect of the tumor on the neurovascular bundle and associated muscle atrophy.

## Figures and Tables

**Table 1:** Types of parosteal lipoma based on degree of chondroid modulation and endochondral ossification [1, 3].

No ossification or chondroid modulation	Lipoma rests directly on the cortex without cartilage or bone elements
Pedunculated exostosis mimic	Narrow bony stalk with a lucent lipomatous cap
Sessile exostosis	Densely ossified broad-based osteochondromatous element beneath the lipomatous cap
Patchy chondroosseous modulation	Foci of calcification, cartilage, or bone throughout the lipomatous mass
